# Efficacy and safety of hyperthermic intraperitoneal chemotherapy in treatment of primary or recurrent ovarian cancer: systematic review and meta-analysis

**DOI:** 10.3389/fmed.2026.1820816

**Published:** 2026-06-11

**Authors:** Abdullah Ali Al Ameer, Shahad Essa Alrehaili, Doaa Wahib Nabulsy, Fatimah Osama Alhamdan, Lina Saleh Aljedani, Dareen Khalid Abdulshafi, Bedoor Obidallah Alghanmi, Abdulrahman Saleh Al-Mulhim

**Affiliations:** 1 Department of Obstetrics and Gynecology, College of Medicine, King Faisal University, Al Ahsa, Saudi Arabia; 2 Department of Obstetrics and Gynecology, College of Medicine, Taibah University, Al Madinah, Saudi Arabia; 3 College of Dentistry, King Abdulaziz University, Jeddah, Saudi Arabia; 4 College of Nursing, Imam Abdulrahman Bin Faisal University, Dammam, Saudi Arabia; 5 Department of General Surgery, College of Medicine, Jeddah University, Jeddah, Saudi Arabia; 6 Department of General Surgery, College of Dentistry, King Abdulaziz University, Jeddah, Saudi Arabia; 7 Department of Oncology, College of Medicine, King Abdulaziz University, Rabigh, Saudi Arabia; 8 Department of General Surgery, College of Medicine, King Faisal University, Al Ahsa, Saudi Arabia

**Keywords:** cytoreductive surgery, epithelial ovarian cancer, hyperthermic intraperitoneal chemotherapy, International Federation of Gynecology and Obstetrics, overall survival, randomized controlled trial

## Abstract

**Background:**

Epithelial ovarian cancer (EOC) is the leading cause of mortality among gynecologic cancers. The standard treatment approach involves primary debulking surgery (PDS) followed by systemic chemotherapy. Hyperthermic intraperitoneal chemotherapy (HIPEC) has emerged as an additional therapeutic option used alongside cytoreductive surgery (CRS); however, its efficacy and safety remain controversial.

**Objective:**

This study aims to compare overall survival (OS) and progression-related outcomes between patients receiving standard multimodal treatment with intraoperative HIPEC and those receiving standard multimodal treatment without HIPEC for primary advanced or recurrent EOC, and to evaluate postoperative complications.

**Methods:**

A systematic search of Ovid-Medline, Web of Science, and PubMed was conducted without date restrictions through February 2025. Only randomized controlled trials (RCTs) were included. Two reviewers independently performed study quality assessments and data extraction. The protocol was registered in PROSPERO (CRD420251067703). Meta-analysis was conducted using fixed- and random-effects models to pool hazard ratios (HR), risk ratios (RR), and mean differences. Heterogeneity was assessed using the I^2^ statistic.

**Results:**

Six RCTs (*n* = 773) with moderate-quality evidence were included; of these, four studies (555 patients) were conducted in primary settings and two (218 patients) in recurrent settings. The meta-analysis showed a significant improvement in OS among patients with primary ovarian cancer receiving HIPEC (HR = 0.74, 95% CI, 0.60 to 0.92), but no improvement in the recurrent setting (HR = 1.08, 95% CI, 0.62 to 1.90). No significant differences were observed in progression-related outcomes or postoperative complications (primary ovarian cancer: HR = 0.71, 95% CI, 0.43 to 1.18; recurrent ovarian cancer: HR = 1.54, 95% CI, 1.00 to 2.37), (RR = 1.08, 95% CI = 0.94 to 1.24, I^2^ = 0.0%), respectively.

**Conclusion:**

HIPEC may benefit selected patients with primary advanced EOC, particularly in trials evaluating its use during interval debulking surgery. However, the limited and heterogeneous RCT evidence warrants cautious interpretation.

**Systematic review registration:**

CRD420251067703 https://www.crd.york.ac.uk/PROSPERO/view/CRD420251067703

## Highlights

- HIPEC may provide a survival benefit in selected patients with primary advanced ovarian cancer.
- HIPEC after neoadjuvant chemotherapy and interval debulking was associated with improved overall survival in primary ovarian cancer.
- HIPEC has no established role in recurrent ovarian cancer.
- HIPEC is a potential safe treatment option for ovarian cancer.

## 1 Introduction

Ovarian cancer is considered the leading cause of death among gynecologic cancers in women (1). Approximately 70% of women with epithelial ovarian cancer (EOC) are diagnosed at an advanced stage (International Federation of Gynecology and Obstetrics (FIGO) stage > II, indicating tumor extension beyond the pelvis), which is associated with a poorer prognosis and high postoperative morbidity (2). The 5-year survival rate is less than 40% when the disease is diagnosed at a regional or distant stage (2).

For advanced ovarian cancer, the standard treatment is primary debulking surgery (PDS), followed, in most cases, by systemic chemotherapy (3). Compared with intravenous (IV) chemotherapy, intra-abdominal chemotherapy allows more direct drug distribution across the peritoneal surface, leading to improved survival outcomes (4). Hyperthermic intraperitoneal chemotherapy (HIPEC) is a form of intra-abdominal chemotherapy administered intraoperatively after heating the chemotherapeutic agents (4). This heating enhances drug efficacy and tissue penetration (4). HIPEC primarily utilizes platinum-based chemotherapeutic agents (4).

A previous systematic review and meta-analysis demonstrated that adding HIPEC to cytoreductive surgery (CRS) improves overall survival (OS) and disease-free survival (DFS) in patients with advanced EOC without increasing complication rates (1). Another systematic review and meta-analysis reported that combining HIPEC with interval debulking surgery (IDS) and neoadjuvant chemotherapy is a safe and effective strategy that significantly improves 5-year OS and DFS in patients with primary ovarian cancer (5).

However, studies by Chiva et al. (6) and Baiocchi et al. (7) suggest that the use of HIPEC in both primary advanced and recurrent ovarian cancer does not provide a clear survival benefit. Consequently, the role of HIPEC in ovarian cancer management remains controversial. Therefore, we conducted a systematic review and meta-analysis to evaluate whether adding intraoperative HIPEC to standard multimodal treatment (CRS combined with systemic chemotherapy) improves survival and postoperative outcomes in patients with primary advanced or recurrent EOC.

A recent systematic review by El Kassis et al. (8) included six randomized controlled trials (RCTs) along with several observational studies and reported improved survival outcomes with HIPEC in the context of IDS. However, the inclusion of non-randomized studies introduces uncertainty due to heterogeneous study designs and potential biases. Therefore, this study was designed to evaluate the efficacy of HIPEC based exclusively on RCTs with the aim of providing the highest level of evidence on this controversial topic.

## 2 Methods

### 2.1 Review of literature

In this study, we followed the Preferred Reporting Items for Systematic Reviews and Meta-Analyses (PRISMA) guidelines. The research protocol was registered in the International Prospective Register of Systematic Reviews (PROSPERO) under the identifier CRD420251067703. A completed PRISMA checklist is provided in Supplementary Table S1.

The literature search was conducted in February 2025 using PubMed, Ovid MEDLINE, and Web of Science. Only English-language studies were included, with no restrictions on publication date.

The search strategy combined Medical Subject Headings (MeSH) and free-text terms related to HIPEC, EOC, CRS, disease setting, survival outcomes, and postoperative complications. Key MeSH terms included “Hyperthermic Intraperitoneal Chemotherapy,” “Ovarian Neoplasms,” “Carcinoma, Ovarian Epithelial,” “Cytoreduction Surgical Procedures,” “Neoplasm Recurrence, Local,” “Survival Analysis,” “Disease-Free Survival,” “Treatment Outcome,” “Postoperative Complications,” and “Randomized Controlled Trial.” The full reproducible search strategies for each database are provided in Supplementary Table S2.

The population, intervention, comparison, outcome, and study design (PICOS) criteria included RCTs evaluating the effect of adding intraoperative HIPEC to standard multimodal treatment on survival, mortality, and morbidity in patients with primary advanced or recurrent EOC compared with those who did not receive HIPEC.

### 2.2 Methodology of selecting studies

The eligibility criteria for study inclusion and exclusion are summarized in Box 1.

**Box 1 Eligibility criteria for study inclusion and exclusion.**

**Table:** 

Inclusion criteria	Exclusion criteria
Adult patients aged ≥18 years with epithelial ovarian cancer	Animal or *in vitro* studies
Primary advanced or recurrent epithelial ovarian cancer	Other gynecological malignancies
Studies evaluating HIPEC as part of ovarian cancer multimodal management	Studies not evaluating HIPEC
Comparator group receiving standard treatment without HIPEC	Studies without a non-HIPEC comparator
Reported at least one relevant survival, morbidity, or postoperative outcome	Studies without relevant extractable outcomes
Randomized controlled trials	Reviews, meta-analyses, editorials, letters, commentaries, case reports, case series, cross-sectional studies
English-language publications	Non-English publications

### 2.3 Outcome measures

The primary outcomes evaluated in this review were OS, DFS, progression-free survival (PFS), and recurrence-free survival (RFS). Secondary outcomes included the proportion of patients experiencing postoperative grade III–IV adverse events, 30-day postoperative mortality, disease progression, duration of follow-up, length of hospital stay (LOS), and the time interval between completion of the initial surgical procedure and initiation of adjuvant chemotherapy.

### 2.4 Process of screening and data extraction

Primary screening of titles and abstracts, followed by full-text screening, was conducted independently by two reviewers using the Rayyan platform. Any disagreements were resolved by a third reviewer.

Data extraction was performed using a standardized data collection form. Extracted information included study-level characteristics, such as the first author’s last name, year of publication, journal name, country of origin, recruitment period, study design, study population, and sample size. It also included patient-related variables, such as age and medical history.

Treatment-related data were extracted, including the number of patients who received HIPEC versus those who did not, as well as details of the HIPEC protocol (e.g., technique, drugs administered, duration, and temperature) and the type of chemotherapy used in both the intervention and control groups. Additionally, information on the surgical approach, categorized as primary or recurrent EOC, was collected.

Disease-related variables included FIGO stage, histological subtype, and the peritoneal carcinomatosis index (PCI). Extracted outcomes included median follow-up, median OS, DFS, PFS, RFS, number of deaths, and number of disease progression events. Additional outcomes included the incidence and severity of grade III or IV adverse events, classified according to the National Cancer Institute Common Terminology Criteria for Adverse Events (CTCAE) version 4.0, as well as LOS and the interval between completion of the initial chemotherapy session and initiation of adjuvant chemotherapy.

### 2.5 Assessment of quality and Bias risk

The Cochrane Risk of Bias 2 (RoB 2) tool was used to assess the methodological quality of the included RCTs. This tool evaluates five domains: (1) bias arising from the randomization process, (2) bias due to deviations from intended intervention, (3) bias due to missing outcome data, (4) bias in measurement of the outcome, and (5) bias in selection of the reported result.

### 2.6 Meta-analysis

As a few RCTs reported each endpoint separately, DFS, PFS, and RFS were pooled as a composite progression-related endpoint to ensure quantitative feasibility. This composite was interpreted cautiously and was not intended to imply clinical equivalence among these endpoints.

Mean and standard deviation values were estimated from reported median and interquartile ranges (9). Time-to-event data were expressed as HRs with 95% confidence intervals (CIs). When HRs were not directly reported, they were estimated from Kaplan–Meier curves using WebPlotDigitizer (version 5.2) (10). Dichotomous outcomes were pooled as risk ratios (RRs) and continuous outcomes as mean differences (MDs), each with 95% CIs. Meta-analyses were conducted using both common-effect and random-effects models, and heterogeneity was assessed using the I^2^ statistic. Publication bias was evaluated using funnel plots and Egger’s test.

The robustness of the pooled estimates was assessed through leave-one-out sensitivity analysis, in which each study was sequentially omitted and the effect size recalculated for all outcomes. All analyses were performed using RStudio (version 2024.9.1.394, Boston, MA, USA) with R version 4.4.2. A two-sided *p*-value < 0.05 was considered statistically significant.

Exploratory univariable meta-regression analyses were conducted to investigate potential sources of heterogeneity in OS and progression-related outcomes. Due to the limited number of studies, each covariate was analyzed separately. The following covariates were examined: disease setting, timing of surgery, HIPEC drug type, HIPEC infusion time, intra-abdominal temperature, HIPEC infusion technique, duration of follow-up, CC-0 cytoreduction rate, and sample size. An R^2^ analog was used to estimate the proportion of heterogeneity explained by each covariate, and the Q residual statistic was used to quantify residual heterogeneity. Given the small number of studies, these analyses were considered exploratory and hypothesis-generating.

## 3 Results

### 3.1 Study screening and inclusion

A total of 5,016 records were identified across the three databases searched, of which 1,136 duplicates were removed. The remaining 3,880 titles and abstracts were screened, and 3,818 records were excluded. Consequently, 62 studies were sought for retrieval; however, 17 could not be retrieved. Full-text screening was conducted for the remaining 45 studies to assess eligibility.

**Figure 1:**
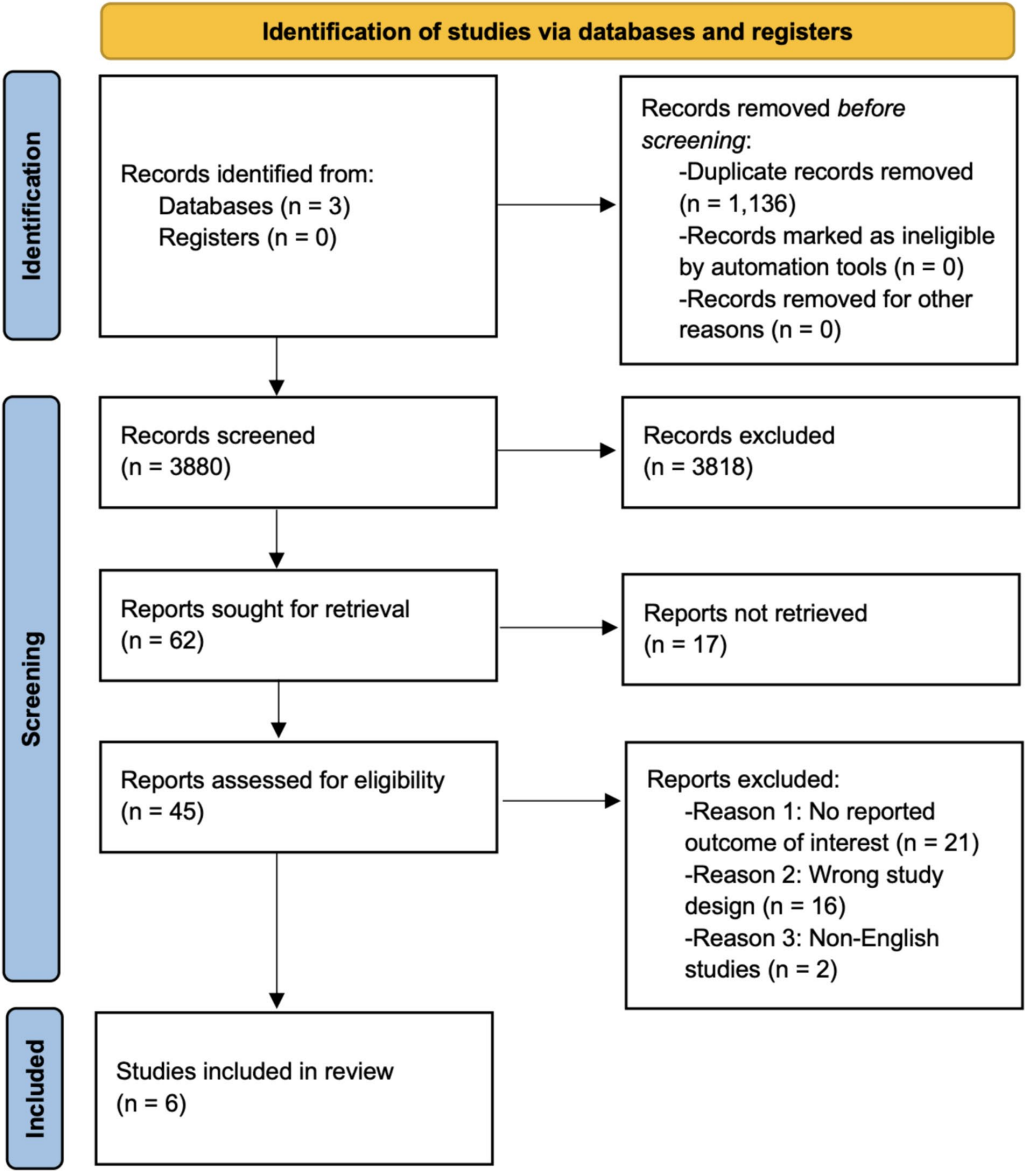
A PRISMA flowchart showing the study screening process.

Based on the predefined inclusion criteria, 21 studies were excluded due to the lack of reported outcomes, 16 due to inadequate study design, and 2 due to being published in non-English languages. Ultimately, six studies were included in the final analysis (11–16) (Figure 1).

### 3.2 Characteristics of clinical trials

A total of six clinical trials, conducted across Europe (11, 12, 14, 15), North America (16), and Asia (13), were included in the analysis. The recruitment periods ranged from 2006 to 2019. Overall, 773 patients were enrolled, with sample sizes ranging from 55 to 245 participants.

Sample sizes for the HIPEC and control groups varied across studies. The largest trial (12) enrolled 122 patients in the HIPEC arm and 123 in the control arm, whereas the smallest trial (15) included 32 and 23 patients in the respective groups. Of the six studies, four (555 patients) were conducted in primary settings and two (218 patients) in recurrent settings.

The median age of participants was generally comparable between the HIPEC and control groups, ranging from 52 to 65.5 years. Most studies focused on advanced-stage disease, with FIGO stages III or IV reported in three of the six trials; however, one study (16) did not specify FIGO stage (Table 1).

**Table 1:** Baseline characteristics of the included randomized controlled trials.

Authors	Country	Journal	Recruitment period (start to end)	Sample size, n (HIPEC/Control)	Age, years, median or mean (HIPEC/Control)	FIGO stage
Primary setting						
Antonio et al. (2022) (11)	Spain	*Society of Surgical Oncology*	March 2012 to November 2018	35/36	56/65.5	III or IV
Aronson et al. (2023) (12)	Netherlands	*Lancet*	April 2007 to April 2016	122/123	61/63	III
Lim et al. (2022) (13)	South Korea	*JAMA surgery*	March 2,2010 to January 22,2016	92/92	52/53.5	III or IV
Spiliotis et al. (2014) (14)	Greece	*Annals of Surgical Oncology*	2006–2013	60/60	Mean 58.3/ Mean 58.1	NA
Villarejo Campos et al. (2024) (15)	Spain	*Current Oncology*	August 2012 to December 2019	32/23	60.34/60.22	II, III, and IV
Recurrent setting						
Spiliotis et al. (2014) (14)	Greece	*Annals of Surgical Oncology*	2006–2013	60/60	Mean 58.3/ Mean 58.1	NA
Zivanovic et al. (2021) (16)	United States	*American Society of Clinical Oncology*	February 2014 to November 2019	49/49	59/58	NA

HIPEC, Hyperthermic Intraperitoneal Chemotherapy; FIGO, International Federation of Gynecology and Obstetrics; HIPEC, hyperthermic intraperitoneal chemotherapy; NA, not available; RCT, randomized controlled trial.

### 3.3 Treatment characteristics and oncologic outcomes

Across the six included studies, high-grade serous carcinoma was the predominant histological subtype in both the HIPEC and control groups. Additional subtypes, such as endometrioid, mucinous, and clear-cell carcinoma, were reported in smaller proportions (12, 13, 15, 16).

PCI scores were reported in most studies, with patients commonly categorized into low, intermediate, and high PCI groups based on cutoffs such as <10 or ≥10 (4, 6–8) (Supplementary Table S1).

HIPEC delivery techniques included both open and closed approaches. The chemotherapeutic agents used during HIPEC varied across studies: cisplatin was the most frequently used (11–15), while other agents included carboplatin (11–13, 15, 16), paclitaxel (11–13, 15), and doxorubicin (14) depending on disease characteristics and platinum sensitivity. HIPEC duration ranged from 60 to 120 min, with temperatures maintained between 40 °C and 43 °C.

In both the HIPEC and control groups, systemic postoperative chemotherapy—typically consisting of carboplatin and paclitaxel—was administered consistently. Thus, the intervention arms generally represented standard treatment comprising CRS and systemic IV chemotherapy with the addition of HIPEC, whereas the control arms received standard treatment without HIPEC.

Median follow-up durations ranged from 32 to 70.8 months. Median OS was higher in the HIPEC groups across nearly all trials, ranging from 26.7 to 69.5 months, compared with 13.4 to 61.3 months in the corresponding control groups (Table 2).

**Table 2:** Treatment characteristics and oncologic outcomes.

Treatment/outcome characteristic and Study group		Lim et al. (2022) (13)	Zivanovic et al. (2021) (16)	Villarejo Campos et al. (2024) (15)	Antonio et al. (2022) (11)	Aronson et al. (2023) (12)	Spiliotis et al. (2014) (14)
HIPEC regimen (the intervention in details)		Cytoreductive surgery, then HIPEC	Secondary cytoreduction followed by (five or six cycles) or secondary cytoreduction with HIPEC	Cytoreductive surgery, then HIPEC	At the end of the cytoreductive surgery, HIPEC was administered	Cytoreductive surgery, then HIPEC	Cytoreductive surgery then HIPEC
HIPEC technique		Closed technique	Closed technique	Closed technique	Open technique (Coliseum)	open technique	40: open, 20: closed
HIPEC drug		Cisplatin 75 m^2^	Carboplatin 800 m^2^	Paclitaxel 175 m^2^	Cisplatin 75 m^2^	cisplatin 100 mg/m^*2^	platinum-sensitive disease (n = 34): cisplatin 100 mg/m^2^ and paclitaxel 175 mg/m^2^; for platinum- resistant disease (n = 26): doxorubicin 35 mg/m^2^ and (paclitaxel 175 mg/m^2^ or mitomycin 15 mg/m^2^)
Duration (min)		90 min	90 min	60 min	60 min	120 min	60 min
Temp (C)		41.5\u00B0C	41c – 43\u00B0C	42–43\u00B0C	42–43\u00B0C	40\u00B0C	42.5\u00B0C
Chemotherapy	*HIPEC group*	6 cycles IV chemo, post op paclitaxel and carboplatin	5 additional cycles of postoperative IV carboplatin-based chemotherapy	Postoperative systemic intravenous (IV) chemotherapy with carboplatin (AUC = 6) and paclitaxel (175 mg/m2) for 6 cycles.	Six cycles of systemic adjuvant chemotherapy were completed per patient with the same carboplatin and paclitaxel scheme.	3 cycles of carboplatin and paclitaxel after surgery.	NR
	*Control group*	6 cycles IV chemo Post op paclitaxel and carboplatin	6 additional cycles of postoperative IV carboplatin-based chemotherapy	Postoperative systemic IV chemotherapy with carboplatin (AUC = 6) and paclitaxel (175 mg/m2) for 6 cycles.	Six cycles of systemic adjuvant chemotherapy were completed per patient with the same carboplatin and paclitaxel scheme.	3 cycles of carboplatin and paclitaxel after surgery.	NR
Median follow-up (IQR), months	*HIPEC group*	69.4 months (55.6–92.1 months)	for the 3 progression-free survivors was 52.8 months, and for the 29 survivors was 39.5 months.	32 months (NR)	32 months (NR)	4.7 years (NR)	NR
	*Control group*	70.8 months (53.6–85.8 months)	for the 11 progression-free survivors was 52.8 months, and for the 32 survivors was 39.5 months	32 months (NR)	32 months (NR)	56.4 months (NR)	NR
Median overall survival (IQR), months	*HIPEC group*	69.5 months (45.6 months to not reported)	52.5 months (NR)	48 months (NR)	52 months (NR)	44.9 months (NR)	26.7 months (NR)
	*Control group*	61.3 months (34.3 months to not reported)	59.7 months (NR)	46 months (NR)	45 months (NR)	33.3 months (NR)	13.4 months (NR)

HIPEC, Hyperthermic Intraperitoneal Chemotherapy; N, Number; NR, Not Reported; OS, Overall Survival; PCI, Peritoneal Carcinomatosis Index; AUC, Area Under the Curve; IV, Intravenous; C, Celsius; CT-PET, Computed Tomography-Positron Emission Tomography.

#### 3.3.1 Survival outcomes (overall survival): primary ovarian cancer

**Figure 2:**
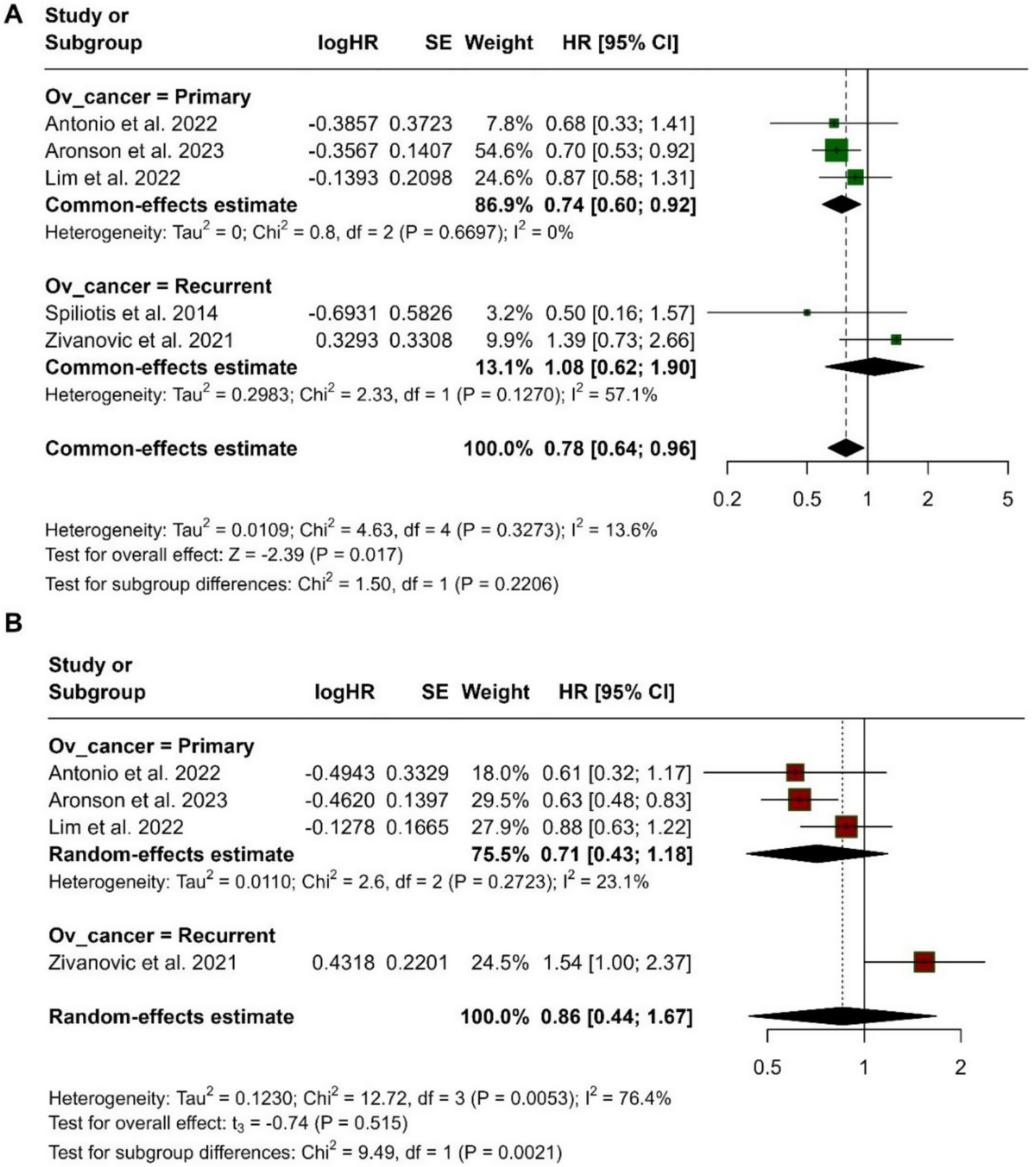
Forest plots of survival outcomes comparing standard treatment plus HIPEC versus standard treatment without HIPEC, stratified by disease setting (primary vs. recurrent). **(A)** For overall survival [primary; number of studies: 3 (*n* = 500) vs. recurrent; number of studies: 2 (*n* = 218)], and **(B)** for progression-related endpoints [primary; number of studies: 3 (*n* = 500) vs. recurrent; number of studies: 1 (*n* = 98)].

Figure 2A presents the HR for OS in the primary ovarian cancer subgroup, which was 0.74 (95% CI, 0.60 to 0.92), indicating a statistically significant survival benefit associated with HIPEC.

**Figure 3:**
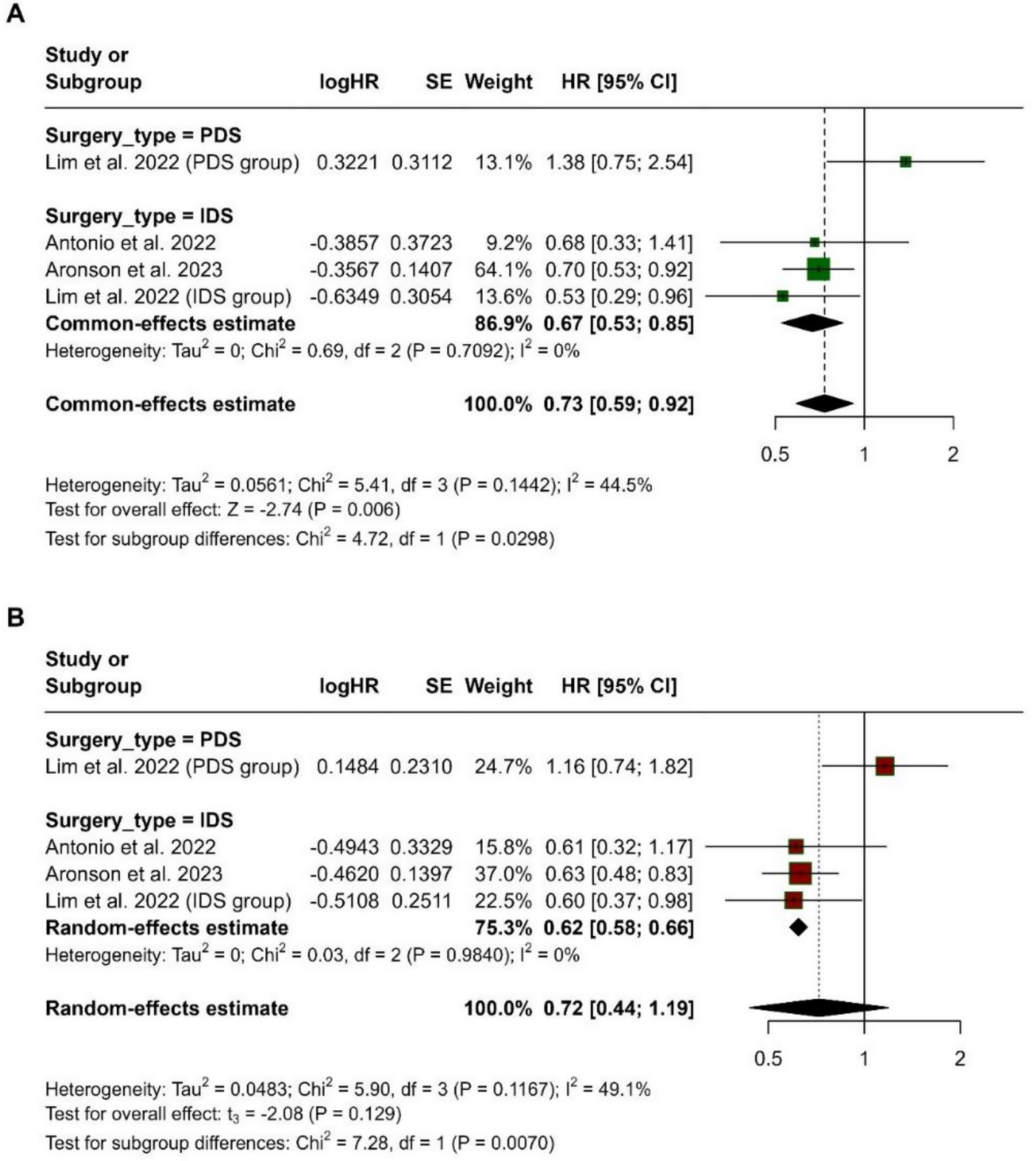
Forest plots of survival outcomes with subgroup analysis of hazard ratios (HRs) by surgery type: comparison between patients undergoing primary debulking surgery (PDS; number of studies: 1 (*n* = 107)) and interval debulking surgery (IDS; number of studies: 3 (*n* = 393)). **(A)** For overall survival, and **(B)** for progression-related endpoints.

In an exploratory subgroup analysis stratified by surgical timing among primary disease trials, the survival benefit associated with HIPEC was primarily observed in studies evaluating its use during IDS, with a pooled HR of 0.67 (95% CI, 0.53 to 0.85). In contrast, no statistically significant benefit was observed in the PDS subgroup, with a pooled HR of 1.38 (95% CI, 0.75 to 2.54). The test for subgroup differences was statistically significant (*p* = 0.030) (Figure 3A).

#### 3.3.2 Survival outcomes (progression-related endpoints): primary ovarian cancer

Figure 2B presents the HR for progression-related endpoints in the primary ovarian cancer subgroup, which was 0.71 (95% CI, 0.43 to 1.18), indicating no statistically significant benefit.

As this composite endpoint combined DFS, PFS, and RFS across trials, the result should be interpreted as a general progression-related signal rather than as a definitive estimate for any individual endpoint.

In an exploratory subgroup analysis stratified by surgical timing, the IDS subgroup showed a significant association between HIPEC and improved progression-related outcomes, with a pooled HR of 0.62 (95% CI, 0.58 to 0.66). In contrast, the PDS subgroup showed no statistically significant effect, with a pooled HR of 1.16 (95% CI, 0.74 to 1.82). The test for subgroup differences was statistically significant (*p* = 0.007), suggesting that the effect of HIPEC varies by surgical context (Figure 3B).

#### 3.3.3 Survival outcomes (overall survival): recurrent ovarian cancer

Figure 2A presents the HR for OS in the recurrent ovarian cancer subgroup, which was 1.08 (95% CI, 0.62 to 1.90), indicating no statistically significant association. The test for subgroup differences between primary and recurrent ovarian cancer subgroups was not statistically significant for OS (*p* = 0.221).

#### 3.3.4 Survival outcomes (progression-related endpoints): recurrent ovarian cancer

Figure 2B presents the HR for progression-related endpoints in the recurrent ovarian cancer subgroup, which was 1.54 (95% CI, 1.00 to 2.37), indicating a borderline statistically significant worsening of outcomes. The test for subgroup differences between primary and recurrent ovarian cancer subgroups was statistically significant (*p* = 0.002), favoring improved outcomes in the primary ovarian cancer subgroup.

### 3.4 Pooled relative risks for postoperative outcomes between the HIPEC and control groups

**Figure 4:**
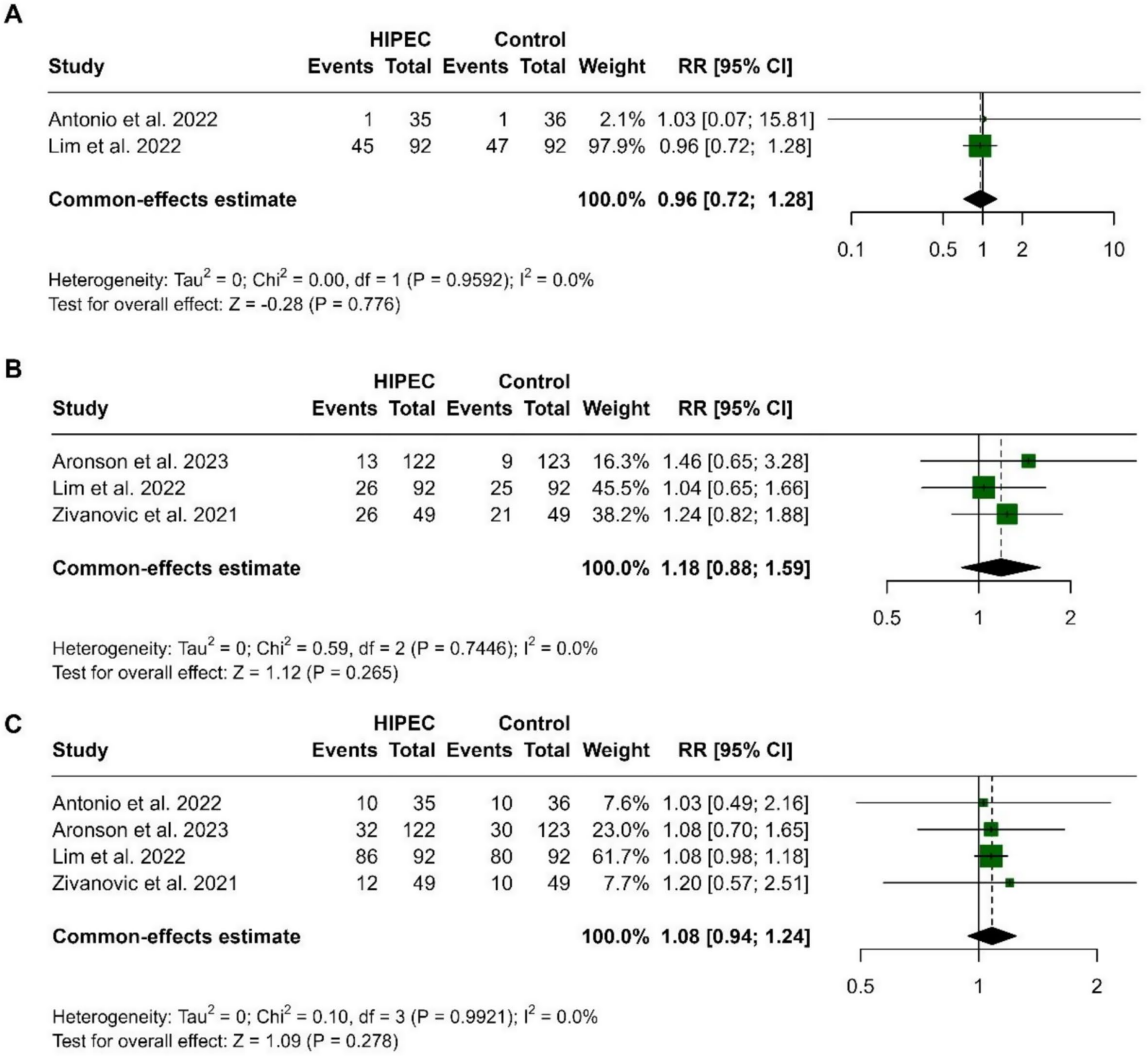
Forest plots depicting the relative risks of 30-day mortality; number of studies: 2 (*n* = 255) **(A)**, disease progression; number of studies: 3 (*n* = 527) **(B)**, and grade III/IV adverse events after surgeries; number of studies: 4 (*n* = 598) **(C)** for standard treatment plus HIPEC versus standard treatment without HIPEC.

For 30-day mortality (Figure 4A), the pooled RR was 0.96 (95% CI, 0.72 to 1.28; *p* = 0.776). For disease progression (Figure 4B), the RR was 1.18 (95% CI, 0.88 to 1.59; *p* = 0.265), and for grade III/IV adverse events (Figure 4C), the RR was 1.08 (95% CI, 0.94 to 1.24; *p* = 0.278). These findings indicate no statistically significant difference between the HIPEC and control groups across these outcomes.

### 3.5 Mean differences in postoperative recovery metrics between the HIPEC and control groups

For LOS (Supplementary Figure S1A), the pooled MD was 1.18 days (95% CI, −1.03 to 3.40; *p* = 0.296). The time between completion of the initial chemotherapy session and initiation of adjuvant chemotherapy (Supplementary Figure S1B) had an MD of 0.32 days (95% CI, −3.33 to 3.98; *p* = 0.862).

### 3.6 Quality assessment

**Figure 5:**
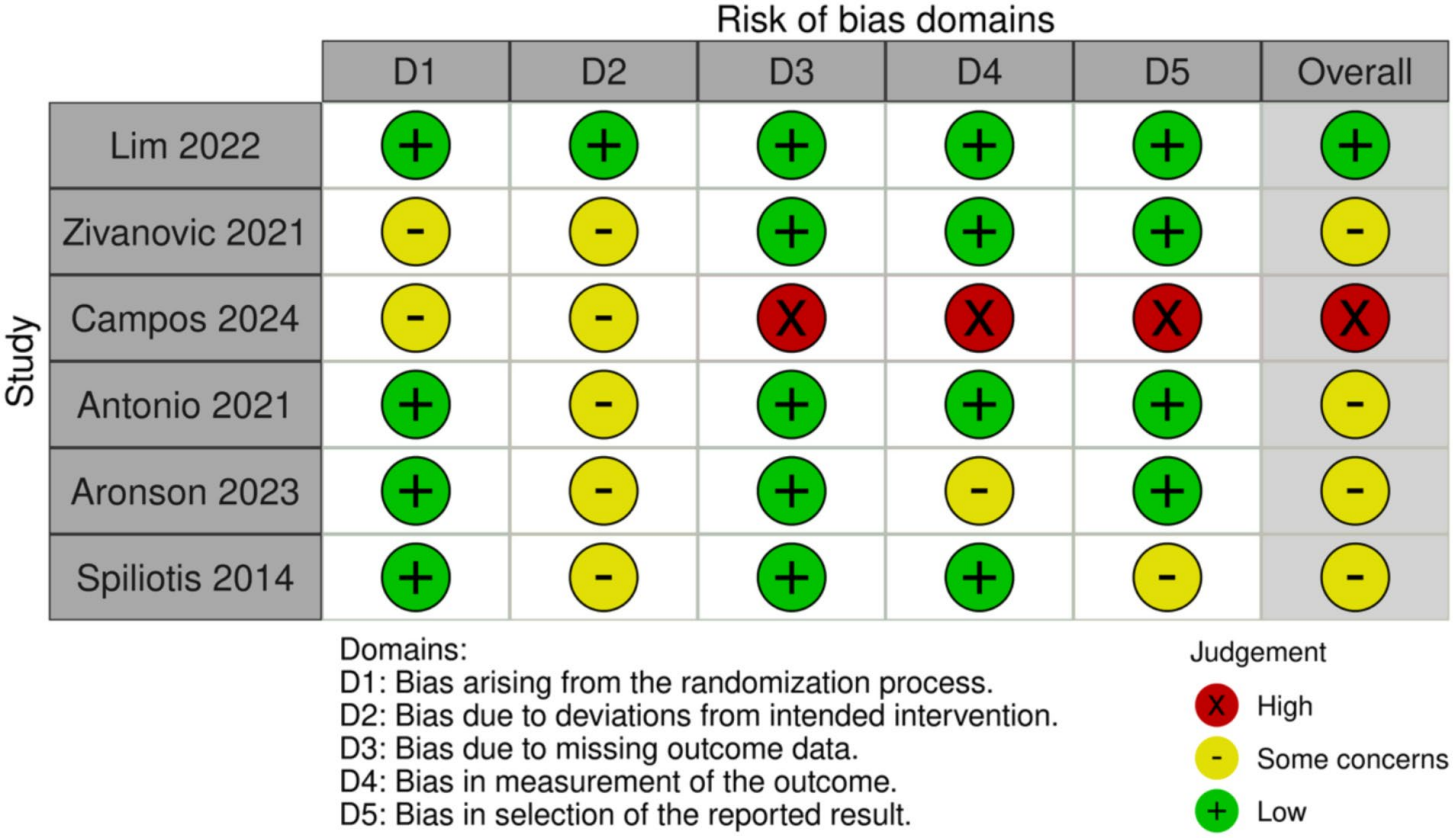
Results of the risk of bias analysis.

Of the six RCTs, one was at low risk of bias (13), four had some concerns (11, 12, 14, 16), and one was at high risk of bias (15) (Figure 5).

### 3.7 Assessment of publication bias

**Figure 6:**
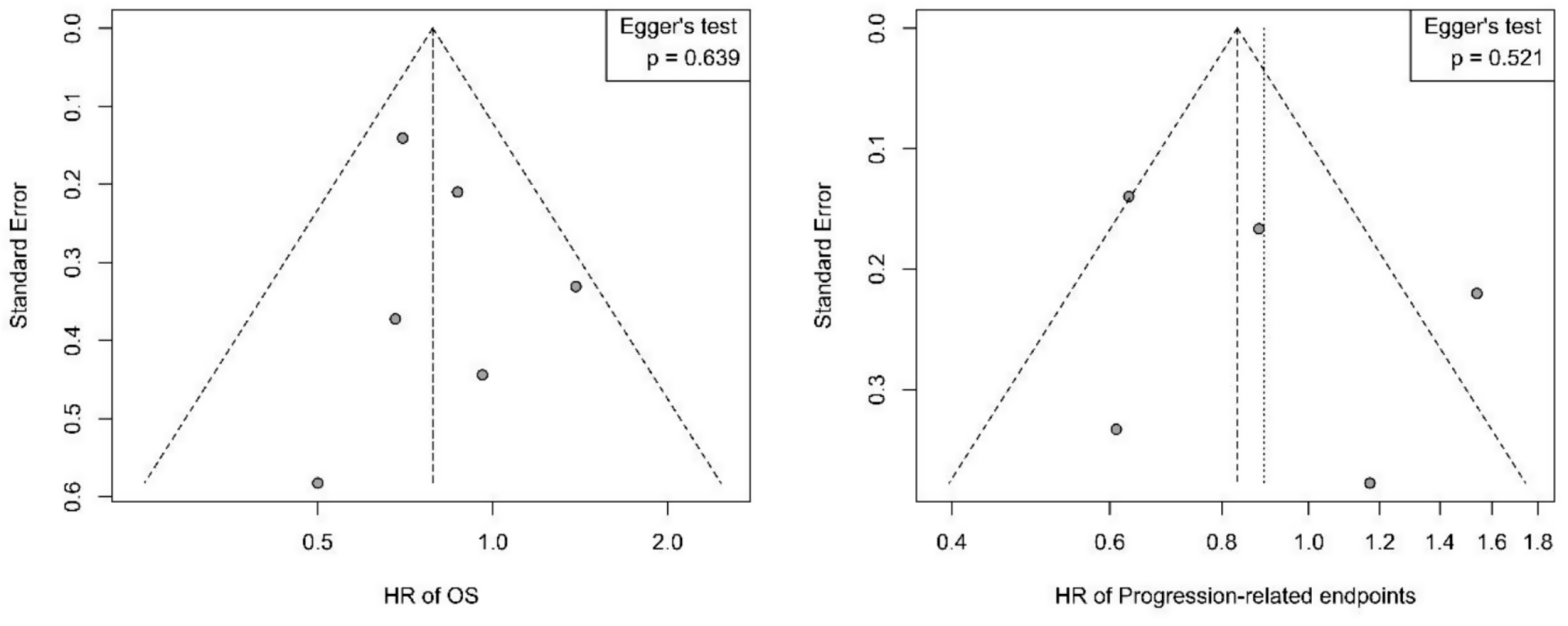
Funnel plots for small-study effects for overall survival and progression-related endpoints.

**Figure 7:**
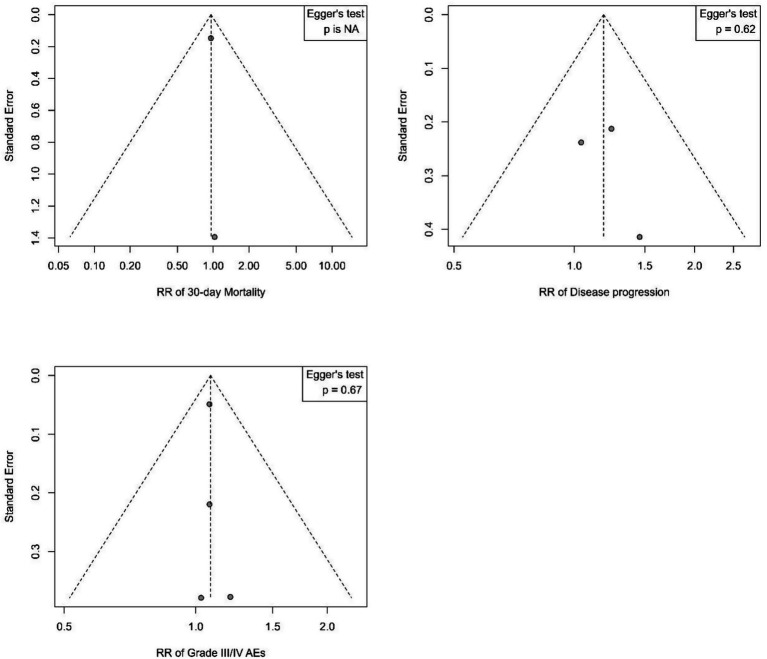
Funnel plots for small-study effects for 30-day mortality, disease progression, and grade III/IV adverse events.

Figures 6, 7 present funnel plots for five outcomes: 30-day mortality, disease progression, grade III/IV adverse events, OS, and progression-related endpoints. LOS and the time between completion of the initial chemotherapy session and initiation of adjuvant chemotherapy are presented in Supplementary Figure S3.

The distribution of studies appears symmetrical across all funnel plots. Egger’s test was not performed for 30-day mortality, LOS, and chemotherapy timing due to the limited number of studies. For disease progression, grade III/IV adverse events, OS, and progression-related endpoints, Egger’s test yielded non-significant results (*p* = 0.62, *p* = 0.67, *p* = 0.639, and *p* = 0.521, respectively), suggesting no evidence of publication bias.

### 3.8 Leave-one-out sensitivity analysis

**Figure 8:**
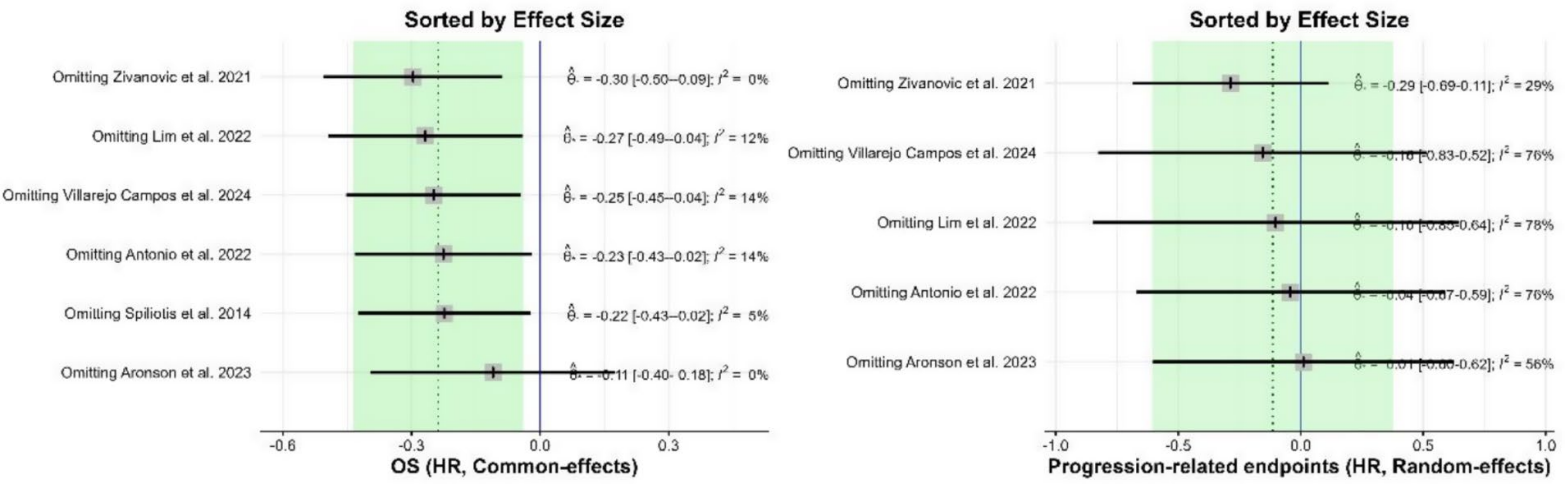
Forest plots showing the results of the leave-one-out sensitivity analysis for time-to-event outcomes [overall survival; number of studies: 6 (*n* = 773) and progression-related endpoints; number of studies: 5 (*n* = 653)].

Figure 8 shows that, for OS, removal of individual studies resulted in only minor changes in the pooled HR. Exclusion of the study by Zivanovic et al. produced the strongest effect, yielding an HR of 0.74 (logHR = −0.30; 95% CI, −0.50 to −0.09), with I^2^ = 0%. The OS benefit of HIPEC remained statistically significant following the removal of each study, except for the study by Aronson et al., which yielded a non-significant result (logHR = −0.11; 95% CI, −0.40 to 0.18) (12, 16).

For progression-related endpoints, exclusion of the study by Zivanovic et al. resulted in a marked reduction in heterogeneity, while the pooled HR remained similar (Figure 8).

**Figure 9:**
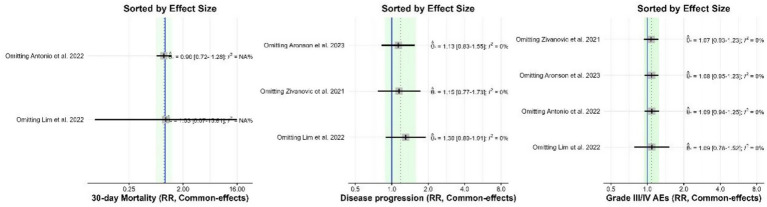
Forest plots showing the results of the leave-one-out sensitivity analysis for categorical outcomes [30-day mortality; number of studies: 2 (*n* = 255), disease progression; number of studies: 3 (*n* = 527), and grade III/IV adverse events; number of studies: 4 (*n* = 598)].

Figure 9 shows that, for 30-day mortality, exclusion of either study (11, 13) did not materially affect the pooled effect size. For disease progression and grade III/IV adverse events, omitting any single study did not change the direction or statistical significance of the pooled relative risks.

For continuous outcomes, including LOS and the time between completion of the initial chemotherapy session and initiation of adjuvant chemotherapy, exclusion of any single study did not meaningfully alter the pooled MD. In all cases, I^2^ remained at 0%, indicating no heterogeneity (Supplementary Figure S3).

### 3.9 Meta-regression analysis

#### 3.9.1 Overall survival

Across the six included studies, the baseline random-effects model yielded a pooled OS HR of 0.755 (95% CI: 0.591–0.966; τ^2^ = 0.032; I^2^ = 35.6%; Q = 7.76, *p* = 0.170). Study-level data used in the meta-regression are presented in Supplementary Table S3, and the results of the nine univariable meta-regression models are shown in Table 3.

**Table 3:** Univariable meta-regression results for overall survival (*k* = 6).

Covariate	HR per unit (95% CI)	95% CI	*p*-value	R^2^ (%)	Q-residual	*p* (Q-residual)
Disease setting (primary = 1, recurrent = 0)	1.063	0.623–1.811	0.823	24.4	4.14	0.265
Surgery timing (IDS = 1, non-IDS = 0)	0.888	0.540–1.462	0.642	34.7	4.26	0.281
Cisplatin use (yes = 1, no = 0)^†^	0.578	0.304–1.099	0.095	100.0	2.19	0.647
HIPEC duration (per 10-min increment)	1.110	0.938–1.314	0.225	100.0	3.49	0.433
HIPEC temperature (°C)	0.932	0.633–1.373	0.722	29.1	4.14	0.272
Infusion technique (open = 1, closed = 0)	0.709	0.409–1.227	0.219	100.0	3.43	0.439
Median follow-up (per 10-month increment)	1.002	0.941–1.066	0.962	21.5	4.09	0.260
CC-0 rate in HIPEC arm (per 10%)	1.096	0.881–1.362	0.411	62.9	3.88	0.331
Sample size (per 10 patients)	0.994	0.959–1.030	0.723	29.0	4.14	0.272

Baseline model (no covariate): pooled OS HR = 0.755 (95% CI: 0.591–0.966); I^2^ = 35.6%; τ^2^ = 0.032. HR per unit = hazard ratio per unit increase in each covariate (or per 10-unit increase for scaled variables). R^2^ = proportion of between-study variance (τ^2^) explained by the covariate. Q-residual = test for residual heterogeneity after inclusion of covariate. ^†^Trend toward significance (*p* < 0.10). CC-0, no visible residual macroscopic disease; CI, confidence interval; HR, hazard ratio; IDS, interval debulking surgery.

Cisplatin-based HIPEC, compared with non-cisplatin regimens (e.g., carboplatin or paclitaxel), showed the strongest signal among the evaluated covariates, with a coefficient HR of 0.578 (95% CI: 0.304–1.099; *p* = 0.095) and an R^2^ of 100%, indicating that cisplatin use fully accounted for the estimated between-study variance in OS. However, this finding did not reach statistical significance at the *α* = 0.05 level.

Similarly, the open infusion technique (HR = 0.709, 95% CI: 0.409–1.227; *p* = 0.219; R^2^ = 100%) and longer HIPEC duration (per 10-min increment, HR = 1.110, 95% CI: 0.938–1.314; *p* = 0.225; R^2^ = 100%) also accounted for all residual τ^2^ but did not reach statistical significance.

Other covariates—including disease setting, surgery timing, HIPEC temperature, follow-up duration, CC-0 rate, and sample size—did not meaningfully explain the observed heterogeneity (R^2^ < 65%; all *p* > 0.21).

#### 3.9.2 Progression-related endpoints

Across the five studies included in the progression-related endpoint analysis, the baseline model demonstrated substantial between-study heterogeneity (pooled HR = 0.864; 95% CI: 0.615–1.214; τ^2^ = 0.093; I^2^ = 67.3%; Q = 12.24, *p* = 0.016). Results of the univariable meta-regression analysis for this outcome are presented in Table 4.

**Table 4:** Univariable meta-regression results for progression-related endpoints (*k* = 5).

Covariate	HR per unit	95% CI	*p*-value	R^2^ (%)	Q-residual	*p* (Q-residual)
Disease setting (primary = 1, recurrent = 0)^†^	0.480	0.209–1.101	0.083	100.0	1.04	0.916
Surgery timing (IDS = 1, non-IDS = 0)	0.614	0.309–1.219	0.164	100.0	1.77	0.665
Cisplatin use (yes = 1, no = 0)	0.586	0.282–1.219	0.153	100.0	1.84	0.689
HIPEC duration (per 10-min increment)	1.076	0.834–1.388	0.573	91.7	4.33	0.362
HIPEC temperature (°C)	1.199	0.664–2.163	0.548	93.6	4.09	0.368
Infusion technique (open = 1, closed = 0)	0.614	0.309–1.219	0.164	100.0	1.77	0.665
Median follow-up (per 10-month increment)	0.951	0.865–1.047	0.306	100.0	2.74	0.481
CC-0 rate in HIPEC arm (per 10%)	0.667	0.390–1.141	0.139	100.0	1.62	0.721
Sample size (per 10 patients)	0.980	0.935–1.027	0.396	100.0	2.48	0.424

Baseline model (no covariate): pooled progression-related HR = 0.864 (95% CI: 0.615–1.214); I^2^ = 67.3%; τ^2^ = 0.093. The five studies contributing to this analysis were Aronson et al. (12), Antonio et al. (11), Lim et al. (13), Villarejo et al. (15), and Zivanovic et al. (16); Spiliotis et al. (14) was excluded because the progression endpoint was not extractable. HR per unit = hazard ratio per unit increase in each covariate (or per 10-unit increase for scaled variables). R^2^ = proportion of between-study variance (τ^2^) explained by the covariate. Q-residual = test for residual heterogeneity after inclusion of covariate. ^†^Trend toward significance (*p* < 0.10). CC-0, no visible residual macroscopic disease; CI, confidence interval; HR, hazard ratio; IDS, interval debulking surgery.

Disease setting (primary vs. recurrent) showed a marginally significant association (HR = 0.480; 95% CI: 0.209–1.101; *p* = 0.083; R^2^ = 100%), suggesting that the direction and magnitude of HIPEC’s effect on progression-related outcomes may differ between primary and recurrent settings.

IDS and cisplatin use each independently explained all residual τ^2^ (R^2^ = 100%), with effect estimates in the protective direction (HRs of 0.614 and 0.586, respectively); however, neither reached statistical significance (both *p* ≈ 0.15–0.16). The open HIPEC technique demonstrated an identical pattern to surgery timing (r = 1.0, collinearity between technique and IDS in this dataset).

No other covariates meaningfully explained the observed heterogeneity in progression-related outcomes.

## 4 Discussion

This meta-analysis evaluates the efficacy and safety of adding HIPEC to standard multimodal treatment, CRS combined with IV chemotherapy, in patients with primary advanced or recurrent ovarian cancer, compared with standard treatment alone. Only RCTs were included, assessing outcomes such as OS, progression-related endpoints, postoperative complications, and recovery metrics.

The analysis included six RCTs comprising a total of 773 patients. Overall, the findings suggest that the addition of HIPEC may improve survival in selected patients with primary ovarian cancer, particularly those undergoing IDS. However, no clear survival benefit was observed in recurrent ovarian cancer, and results for progression-related outcomes were inconsistent. The available RCT data did not demonstrate a statistically significant increase in 30-day mortality, grade III/IV adverse events, LOS, or delay in initiation of adjuvant chemotherapy. However, these safety findings should be interpreted with caution, given the small number of trials, low event rates, and variability in HIPEC protocols (1, 2, 5, 17–23).

We observed a statistically significant improvement in OS associated with the addition of HIPEC in patients with primary ovarian cancer. This finding is supported by multiple studies demonstrating a beneficial effect of HIPEC in this population (1, 5, 23). However, no significant benefit was observed for progression-related endpoints, consistent with the findings of Marzuqi et al. (24).

In recurrent ovarian cancer, no clear survival advantage was observed with the addition of HIPEC to secondary cytoreduction. However, this finding should be interpreted with caution, as recurrent ovarian cancer is a heterogeneous disease, and the effect of HIPEC may vary depending on factors such as platinum sensitivity, response to second-line chemotherapy, site of recurrence, completeness of cytoreduction, and patient selection. In the study by Zivanovic et al., HIPEC with carboplatin during secondary cytoreduction for platinum-sensitive recurrent ovarian cancer was well tolerated but did not confer a survival benefit, suggesting that this specific agent and treatment context may not be effective (16). More recent evidence from the CHIPOR trial provides a contrasting perspective. In patients with platinum-sensitive recurrent ovarian cancer experiencing a first recurrence, who responded to platinum-based chemotherapy and underwent complete secondary cytoreduction, cisplatin-based HIPEC was associated with improved OS but also with an increase in the number of grade ≥3 adverse events (25). Taken together, these findings suggest that the role of HIPEC in recurrent ovarian cancer is context-dependent rather than uniformly ineffective. Its benefit appears to depend on factors such as platinum sensitivity, timing relative to systemic therapy, HIPEC agent, and the achievement of complete cytoreduction.

The observed difference between the IDS and PDS subgroups is clinically meaningful but requires careful interpretation. In our study, the survival benefit associated with HIPEC was more pronounced in trials where HIPEC was administered during IDS, whereas no such benefit was observed in the PDS subgroup. This finding is consistent with previous meta-analyses, including those by El Kassis et al. and Filis et al., which reported the most consistent survival benefit of HIPEC when delivered during interval CRS following neoadjuvant chemotherapy (5, 18). However, our study did not compare IDS and PDS as alternative surgical strategies; rather, it examined whether the effect of adding HIPEC differed across trials conducted in different surgical contexts. Therefore, the observed benefit in the IDS subgroup should be interpreted as a context-specific effect of HIPEC rather than evidence that IDS is superior to PDS. This distinction is particularly important in light of the TRUST trial, which did not meet its primary endpoint of improved OS for primary CRS compared with interval CRS, but did report a PFS advantage for primary surgery in appropriately selected patients treated at expert centers (26). Accordingly, the timing of surgery should be determined on an individual basis, taking into account factors such as resectability, patient performance status, disease burden, institutional expertise, and multidisciplinary evaluation.

In this context, the ongoing OVHIPEC-2 trial is particularly relevant because it directly evaluates whether adding HIPEC to primary CRS improves OS in newly diagnosed FIGO stage III EOC after complete or near-complete primary cytoreduction. This trial is expected to clarify whether the survival benefit of HIPEC, primarily observed in the IDS setting, can be extended to the PDS setting (27).

The exploratory meta-regression analyses provided additional insight into the observed heterogeneity. For OS, cisplatin-based HIPEC showed the strongest trend toward explaining between study-variability, while the open technique and longer HIPEC duration also accounted for residual variability; however, these associations were not statistically significant. For progression-related outcomes, there was a trend suggesting that disease setting, as well as the protective effects of both IDS-based timing and cisplatin use, may contribute to variability in treatment response. These findings indicate that the effectiveness of HIPEC may be influenced by clinical and treatment-related factors, including disease setting, timing of cytoreduction, and choice of HIPEC agent. However, all analyses were univariable, and the small number of included studies (5, 6) limits the reliability of these findings as predictors of treatment benefit.

Studies by El Kassis et al., Llueca et al., and Bouchard-Fortier et al. reported favorable outcomes supporting the use of HIPEC in the management of ovarian cancer (1, 17, 18). However, these effect estimates may be inflated compared with our findings (HR = 0.78), which are based exclusively on RCTs and therefore provide more robust evidence. For example, El Kassis et al. (HR = 0.65) and Llueca et al. (HR = 0.56) reported stronger effect sizes (1, 18). This apparent inflation may be attributed to the inclusion of mixed study designs, which should be interpreted with caution. However, Llueca et al. conducted a subgroup analysis restricted to RCTs, which implied the reliability of their findings (1). Moreover, the inclusion of mixed study designs increases the risk of confounding and bias, thereby affecting the reliability of the results.

The pooled analysis showed no statistically significant difference between the HIPEC and control groups in 30-day postoperative mortality. Similarly, the systematic review and meta-analysis by Della Corte et al. reported 0% 30-day mortality in most included studies, with no increase in perioperative mortality risk in either group (2), supporting the safety profile of HIPEC.

Based on our results, HIPEC did not demonstrate a clear benefit in composite progression-related survival outcomes. This is consistent with the findings of Filis et al., who, in their meta-analysis of RCTs, concluded that HIPEC does not significantly increase the risk of severe postoperative complications (5). Kireeva et al., in their systematic review and meta-analysis, reported variable disease progression rates across studies but noted that progression was generally lower in patients treated with HIPEC following neoadjuvant chemotherapy (22). Progression-related outcomes should be interpreted with caution. HIPEC did not show a consistent effect on pooled progression-related endpoints, which combined DFS, PFS, and RFS. However, the number of RCTs reporting each endpoint individually was insufficient to permit separate meta-analyses. Although related, these endpoints are not equivalent, as they differ in definition, censoring, timing, and interpretation. Therefore, the findings should be considered an overall indication of progression-related disease control rather than precise estimates of DFS, PFS, or RFS. Future trials and meta-analyses should report and analyze these endpoints separately when data are available.

Regarding grade III/IV adverse events, our analysis found no statistically significant difference between HIPEC plus standard treatment and standard treatment alone. Similar findings were reported by two systematic reviews and meta-analyses, Filis et al. and Llueca et al., which also showed no significant difference between the two groups (1, 5). However, because the available RCT evidence remains limited and HIPEC protocols vary across studies, these findings should be interpreted as reassuring but not definitive evidence of safety.

Based on the pooled MD of 1.18 days, the results indicate that HIPEC does not significantly prolong hospital stay. Tsolakidis et al., in their narrative review, similarly reported that HIPEC does not delay recovery or compromise perioperative outcomes (21). Conversely, another study reported longer hospital stays, which may be attributable to center-specific protocols (20).

Our study found no significant difference between the two groups in the time from completion of the initial chemotherapy session to initiation of adjuvant chemotherapy. A previous meta-analysis by Guerra et al. reported similar findings (28).

HIPEC is biologically appealing in ovarian cancer because EOC commonly disseminates along the peritoneal surface, allowing heated intraperitoneal chemotherapy to target residual microscopic disease while limiting systemic exposure. This rationale supports its evaluation as an adjunct to CRS, particularly in carefully selected patients (17).

The potential benefit of HIPEC is often overshadowed by concerns regarding toxicity and increased complication rates, which may contribute to higher morbidity and mortality (5). However, previous studies involving 617 randomized patients suggested that HIPEC is a safe therapeutic option across various ovarian cancer settings (5). Compared with CRS without HIPEC, the incidence of grade ≥ 3 adverse events and early mortality appears similar (5).

According to the 2025 National Comprehensive Cancer Network (NCCN) guidelines for ovarian cancer management, the standard treatment for primary advanced ovarian cancer is upfront PDS, followed in most (but not all) patients, by systemic chemotherapy (3). Adjuvant chemotherapy followed by IDS should be considered for patients who are not suitable candidates for PDS due to advanced age, frailty, poor performance status, comorbidities, or already having diseases that are unlikely to be optimally resected (3). For recurrent ovarian cancer, systemic chemotherapy remains the mainstay of treatment. Secondary CRS may be considered in selected patients, as it can provide a survival benefit (3).

Our analysis suggested that the survival benefit associated with HIPEC was more pronounced in studies where it was delivered during IDS than during primary PDS. This observation differs from the aforementioned guidelines and highlights the need for further research directly comparing these surgical contexts. This finding may be attributable to the additional chemical cytoreductive effect of HIPEC when administered in the IDS setting, which could contribute to improved survival outcomes.

Guidelines also recommend HIPEC plus IV chemotherapy as an option, rather than IV chemotherapy alone, for patients with optimally debulked (<1 cm residual) stage III disease (3). Additionally, HIPEC is not recommended for stages I or IV, as there is insufficient evidence supporting its benefits in these settings (3). Our analysis showed improved OS with HIPEC, particularly in advanced-stage disease (stage III and IV). These findings are broadly consistent with guideline recommendations supporting the addition of HIPEC in selected patients with primary ovarian cancer to improve survival outcomes.

The combined regimen may be associated with increased toxicity compared with IV chemotherapy alone, which should be considered when selecting patients, particularly those with normal kidney function and pre-existing neuropathy (3). Therefore, all patients should be counseled regarding the potential risks and clinical benefits of this approach before undergoing treatment (3). In the present analysis, HIPEC was not associated with a statistically significant increase in major postoperative complications compared with control treatment. These findings support the feasibility of HIPEC in selected patients; however, careful patient selection and the availability of more standardized safety data remain necessary (3).

Our analysis showed no survival benefit associated with adding HIPEC to the treatment of recurrent ovarian cancer, and current guidelines did not specifically address its use in this setting (3).

The British Gynecological Cancer Society (BGCS) guidelines are generally consistent with the NCCN recommendations; however, they do not explicitly specify conditions (such as patient comorbidities) for selecting between PDS and IDS (29).

### 4.1 Strengths

Based on our meta-analysis of six RCTs, this study provides a high level of evidence regarding the use of HIPEC in the first-line treatment of ovarian cancer. Several of the included studies were published recently: Antonio et al. (11) and Zivanovic et al. (16) in 2021, Lim et al. (13) in 2022, Aronson et al. (12) in 2023, Villarejo Campos et al. (15) in 2024, and Spiliotis et al. (14) in 2014. Additionally, the inclusion of a large combined sample size of more than 770 patients enhances the robustness and reliability of the findings. The study also evaluated a broad range of outcomes, including progression-related endpoints (DFS, RFS), 30-day mortality, disease progression, and postoperative recovery metrics, providing a comprehensive assessment of HIPEC. Finally, survival outcomes for primary and recurrent ovarian cancer were analyzed separately through subgroup analysis, allowing for more precise interpretation by considering these as distinct clinical entities.

### 4.2 Limitations

Our study has two main limitations. First, the included studies exhibited variability in treatment methodologies. For example, they used different chemotherapeutic agents (cisplatin, carboplatin, paclitaxel, and doxorubicin), temperatures (40–43 °C), durations (60–120 min), and delivery methods (open vs. closed). In addition, patients differed in baseline disease characteristics (e.g., PCI) and were treated across centers with varied levels of expertise. These factors likely contributed to the observed heterogeneity and the modest OS benefit. Although we applied a random-effects model and conducted sensitivity analysis to address this variability, the limited number of studies restricted the effectiveness of these approaches. Second, the methodological quality of the included trials was variable: one study was assessed as low risk of bias, four as moderate risk, and one as high risk. This distribution may compromise the internal validity of individual studies and, consequently, the reliability of the findings. To address this, we performed leave-one-out sensitivity analysis, which showed no significant change in OS (the primary outcome) following removal of individual studies, except for the large and precise study by Aronson et al. (12). Nevertheless, the limitations inherent in the primary data cannot be fully eliminated. The findings for setting-specific analyses should be interpreted with caution due to the limited number of RCTs available. Additionally, the lack of reporting and heterogeneity of study-level baseline data limited the ability to conduct a more detailed analysis of cross-trial comparability. Additionally, the assessment of publication bias was limited by the small number of studies per outcome; therefore, funnel plots and Egger’s tests should be considered exploratory and cannot reliably exclude publication bias. The use of composite progression-related endpoints (DFS, PFS, RFS) may also introduce interpretive limitations, as these outcomes are not fully equivalent. Furthermore, the meta-regression analyses were constrained by the small number of studies, reliance on study-level rather than patient-level variables, potential collinearities among covariates (e.g., IDS timing and open technique), and the inability to perform robust multivariable analyses. Accordingly, these findings should be regarded as exploratory. Overall, these limitations necessitate cautious interpretation of the results. The observed OS benefit in primary ovarian cancer should be considered hypothesis-generating rather than definitive, and the absence of a clear increase in toxicity should not be interpreted as conclusive evidence of safety. Future RCTs are needed to standardize HIPEC protocols and improve methodological quality to generate more reliable and robust evidence.

## 5 Conclusion

This meta-analysis of only RCTs indicates that HIPEC, when added to standard multimodal therapy, may improve OS in a subset of patients with primary advanced ovarian cancer, with signals of greater benefit in the interval debulking setting. No clear effect was observed in recurrent disease, and progression-related outcomes were not consistently improved. Although HIPEC did not significantly increase major postoperative complications in the included trials, the evidence regarding safety remains limited due to the small number of studies and variability in treatment protocols. Well-designed, adequately powered RCTs are needed to identify optimal patient selection, timing, treatment regimen, and safety of HIPEC prior to widespread adoption.

## Data Availability

The datasets presented in this article are not readily available because English language articles only. Requests to access the datasets should be directed to Pubmed, web of science, OVID medline.

## Glossary

BGCS: British Gynaecological Cancer Society
CI: Confidence interval
CRS: Cytoreductive surgery
CTCAE: Common Terminology Criteria for Adverse Events
DFS: Disease-free survival
EOC: Epithelial ovarian cancer
FIGO: International Federation of Gynecology and Obstetrics
HIPEC: Hyperthermic intraperitoneal chemotherapy
HR: Hazard ratio
IDS: Interval debulking surgery
IQR: Interquartile range
IV: Intravenous
LOS: Length of hospital stay
MD: Mean difference
MeSH: Medical Subject Headings
NCCN: National Comprehensive Cancer Network
NR: Not reported
OS: Overall survival
PCI: Peritoneal carcinomatosis index
PDS: Primary debulking surgery
PFS: Progression-free survival
PICOS: Population, Intervention, Comparator, Outcomes, and Study design
PRISMA: Preferred Reporting Items for Systematic Reviews and Meta-Analyses
PROSPERO: International Prospective Register of Systematic Reviews
RCT: Randomized controlled trial
RFS: Recurrence-free survival
RoB 2: Revised Cochrane risk-of-bias tool for randomized trials
RR: Risk ratio
