# Supplementary material for: Efficacy and safety of hyperthermic intraperitoneal chemotherapy in treatment of primary or recurrent ovarian cancer: systematic review and meta-analysis
Source: Front Med (Lausanne). 2026 Jun 11;13:1820816. doi: 10.3389/fmed.2026.1820816 (PMC13294293; doi:10.3389/fmed.2026.1820816)
Supplement: Supplementary file 2 [file Table_2.DOCX]

Table S2. Search strategy for each database

| **Database** | **Search strategy** |
| --- | --- |
| **PubMed** | ("Hyperthermic Intraperitoneal Chemotherapy"[Mesh] OR HIPEC[tiab] OR "hyperthermic intraperitoneal chemotherapy"[tiab] OR "heated intraperitoneal chemotherapy"[tiab] OR "hyperthermic intraperitoneal perfusion"[tiab] OR "hyperthermic intraperitoneal chemoperfusion"[tiab] OR "intraoperative hyperthermic chemotherapy"[tiab] OR "continuous hyperthermic peritoneal perfusion"[tiab]) AND ("Ovarian Neoplasms"[Mesh] OR "Carcinoma, Ovarian Epithelial"[Mesh] OR "ovarian cancer"[tiab] OR "ovarian carcinoma"[tiab] OR "ovarian neoplasm"[tiab] OR "ovarian neoplasms"[tiab] OR "epithelial ovarian cancer"[tiab]) AND (randomized controlled trial[pt] OR controlled clinical trial[pt] OR randomized[tiab] OR randomised[tiab] OR randomly[tiab] OR trial[tiab]) NOT (animals[mh] NOT humans[mh]) |
| **Ovid MEDLINE** | 1. exp Hyperthermic Intraperitoneal Chemotherapy/ OR HIPEC.mp. OR hyperthermic intraperitoneal chemotherap*.mp. OR heated intraperitoneal chemotherap*.mp. OR hyperthermic intraperitoneal perfusion.mp. OR hyperthermic intraperitoneal chemoperfusion.mp. OR intraoperative hyperthermic chemotherap*.mp. OR continuous hyperthermic peritoneal perfusion.mp.  2. exp Ovarian Neoplasms/ OR exp Carcinoma, Ovarian Epithelial/ OR ovarian cancer*.mp. OR ovarian carcinoma*.mp. OR ovarian neoplasm*.mp. OR epithelial ovarian cancer*.mp.  3. randomized controlled trial.pt. OR controlled clinical trial.pt. OR random*.ti,ab. OR trial.ti,ab.  4. 1 AND 2 AND 3  5. limit 4 to humans  6. limit 5 to English language |
| **Web of Science** | TS=(HIPEC OR "hyperthermic intraperitoneal chemotherapy" OR "heated intraperitoneal chemotherapy" OR "hyperthermic intraperitoneal perfusion" OR "hyperthermic intraperitoneal chemoperfusion" OR "intraoperative hyperthermic chemotherapy" OR "continuous hyperthermic peritoneal perfusion") AND TS=("ovarian cancer" OR "ovarian carcinoma" OR "ovarian neoplasm*" OR "epithelial ovarian cancer") AND TS=(random* OR randomised OR randomized OR trial OR "controlled trial" OR "clinical trial") |
