# Supplementary material for: Efficacy and safety of hyperthermic intraperitoneal chemotherapy in treatment of primary or recurrent ovarian cancer: systematic review and meta-analysis
Source: Front Med (Lausanne). 2026 Jun 11;13:1820816. doi: 10.3389/fmed.2026.1820816 (PMC13294293; doi:10.3389/fmed.2026.1820816)
Supplement: Supplementary file 3 [file Table_3.DOCX]

**Supplementary Table 3.** Study-Level Data Used in Meta-Regression Analysis for Overall Survival

| **Study** | **Setting** | **HR*OS* (95% CI)** | **HR Source** | **Surgery Type** | **HIPEC Drug** | **Duration (min)** | **Temp (°C)** | **CC-0 Rate HIPEC (%)** |
| --- | --- | --- | --- | --- | --- | --- | --- | --- |
| Aronson 2023 | Primary | 0.70 (0.53–0.92) | Reported | IDS | Cisplatin 100 mg/m² | 90 | 40–42 | 95 |
| Antonio 2022 | Primary | 0.72 (0.38–1.35)† | Estimated | IDS | Cisplatin 75 mg/m² | 60 | 42–43 | 94 |
| Lim 2022 | Primary | 0.87 (0.58–1.32) | Reported | PDS + IDS | Cisplatin 75 mg/m² | 90 | 41.5 | 82 |
| Villarejo 2024 | Primary | 0.91 (0.38–2.20)† | Estimated | PDS + IDS + Secondary | Paclitaxel 175 mg/m² | 60 | 42–43 | 95 |
| Spiliotis 2015 | Recurrent | 0.50 (0.33–0.77)† | Estimated | Secondary | Cisplatin/Doxorubicin ± Paclitaxel* | 60 | 42.5 | 65 |
| Zivanovic 2021 | Recurrent | 1.39 (0.73–2.66) | Reported | Secondary | Carboplatin 800 mg/m² | 90 | 41–43 | 82 |

*† HR estimated from Kaplan–Meier curves via WebPlotDigitizer; 95% CI approximated from observed event counts. *Spiliotis used cisplatin 100 mg/m² + paclitaxel 175 mg/m² for platinum-sensitive disease, and doxorubicin 35 mg/m² + paclitaxel 175 mg/m² or mitomycin 15 mg/m² for platinum-resistant disease. Abbreviations: CI, confidence interval; CC-0, no visible residual macroscopic disease; HR, hazard ratio; IDS, interval debulking surgery; PDS, primary debulking surgery.*
