# Supplementary material for: Efficacy and safety of hyperthermic intraperitoneal chemotherapy in treatment of primary or recurrent ovarian cancer: systematic review and meta-analysis
Source: Front Med (Lausanne). 2026 Jun 11;13:1820816. doi: 10.3389/fmed.2026.1820816 (PMC13294293; doi:10.3389/fmed.2026.1820816)
Supplement: Supplementary file 4 [file Table_4.DOCX]

**Supplementary Materials**

**Table S4.** Histological subtypes and Peritoneal carcinomatosis index score

|  | **Author** | **Lim et al. 2022** | **Zivanovic et al. 2021** | **Villarejo Campos et al. 2024** | **Antonio et al. 2022** | **Aronson et al. 2023** | **Spiliotis et al. 2014** |
| --- | --- | --- | --- | --- | --- | --- | --- |
| Histological subtypes (Number of patients) | HIPEC group | Serous (N=85), Endometrioid (N=3), Clear cell (N=0), Others (N=4) | High-grade serous histology (N=47) | Serous(N=24), Mucinous (N=3), Endometrioid (N=2), Clear-cell (N=2), Other (1) | NR | High-grade serous (112) High-grade endometrioid (1) Carcinosarcoma (1) Mucinous (1) Clear-cell carcinoma (0) Low-grade serous (4) Low-grade endometrioid (2) Metastasis of gastrointestinal tumor(0) Unknown (1) | NR |
|  | Control group | Serous (N=79), Endometrioid (N=5), Clear cell (N=4), Others (N=4) | High-grade serous histology (N=48) | Serous(N=16), Mucinous (N=1), Endometrioid (N=2), Clear-cell (N=3), Other (N=1) | NR | High-grade serous (107) High-grade endometrioid (1) Carcinosarcoma (4) Mucinous (2) Clear-cell carcinoma (5) Low-grade serous (2) Low-grade endometrioid (0) Metastasis of gastrointestinal tumor(1) Unknown (1) | NR |
| Peritoneal carcinomatosis index score | HIPEC group | 0-5 score (N=22) , 6-10 score (N=70) | NR | CT-PET PCI: <10 score (N=19), 11–20 score (N=11), >20 score (N=1). Surgery PCI: <10 score (N=22), 11–20 score (N=8), >20 score (N=2). | Median (range):10 (2–22) | NR | (PCI < 5: 7), (PCI greater than or equal to 5 OR greater than 10: 24), (PCI greater than or equal to 10: 29) |
|  | Control group | 0-5 score (N=29) , 6-10 score (N=63) | NR | CT-PET PCI: <10 score (N=14), 11–20 score (N=7), >20 score (N=2). Surgery PCI: <10 score (N=14), 11–20 score (N=8), >20 score (N=1). | Median (range):7 (2–29) | NR | (PCI < 5: 8), (PCI greater than or equal to 5 OR greater than 10: 22), (PCI greater than or equal to 10: 30) |

*Abbreviations: HIPEC: Hyperthermic Intraperitoneal Chemotherapy, N: Number, NR: Not Reported, PCI: Peritoneal Carcinomatosis Index, CT-PET: Computed Tomography-Positron Emission Tomography.*


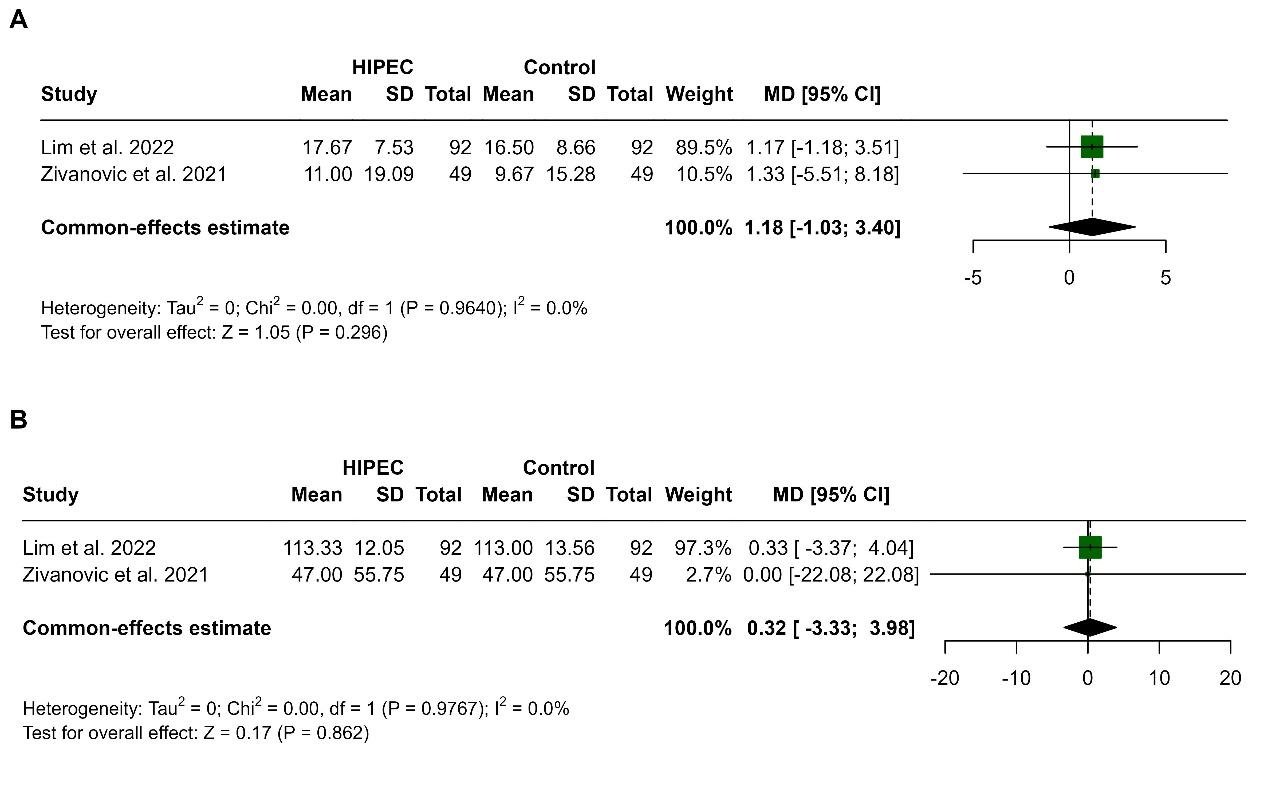


**Figure S1.** Forest plots depicting the mean differences in the length of hospital stay(A) and the time between end of first chemotherapy session and initiation of the adjuvant chemotherapy(B); number of studies: 2 (n=282).


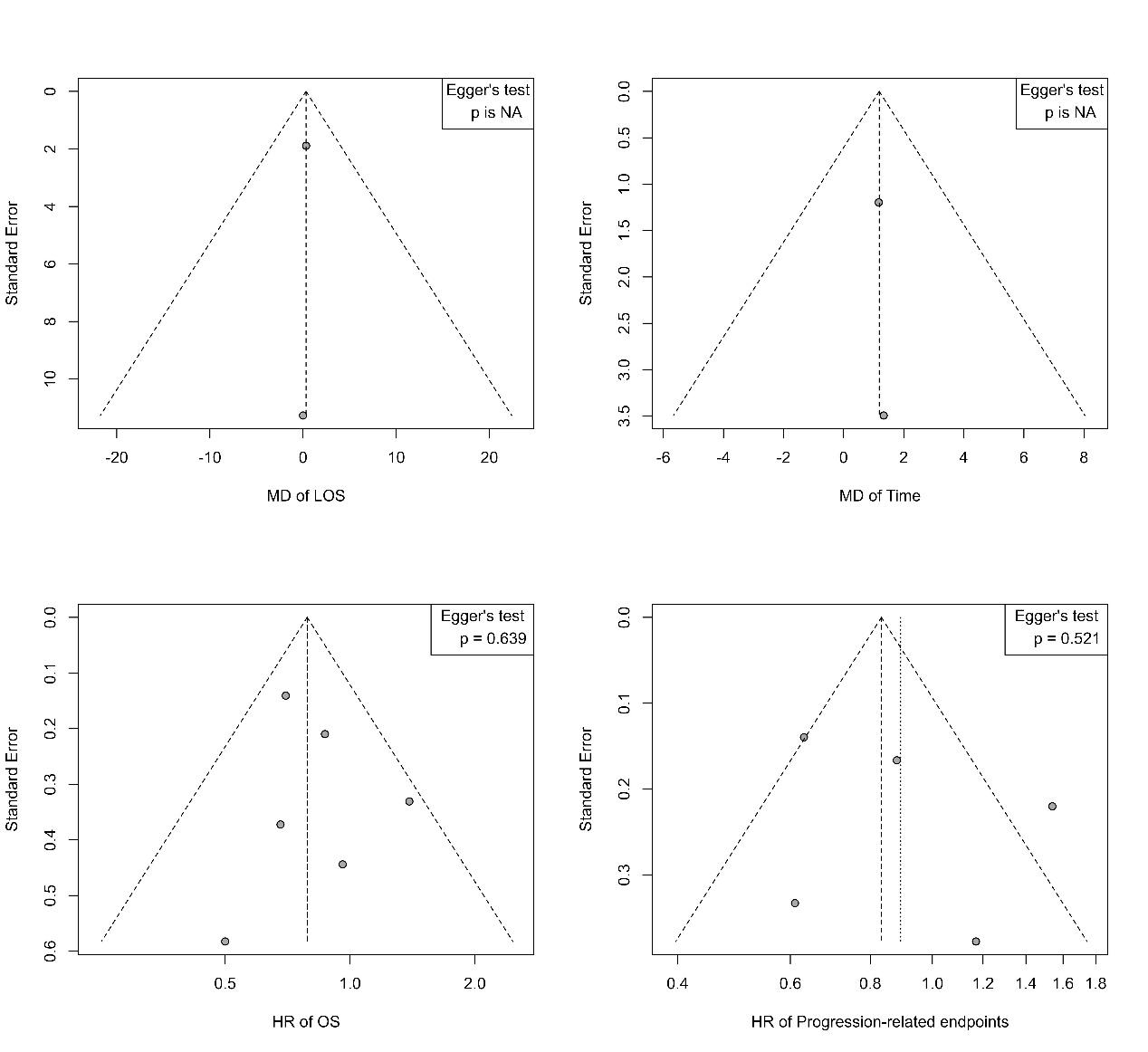


**Figure S2.** Funnel plots showing the risk of publication bias for the length of hospital stay and the time between end of first chemotherapy session and initiation of the adjuvant chemotherapy.


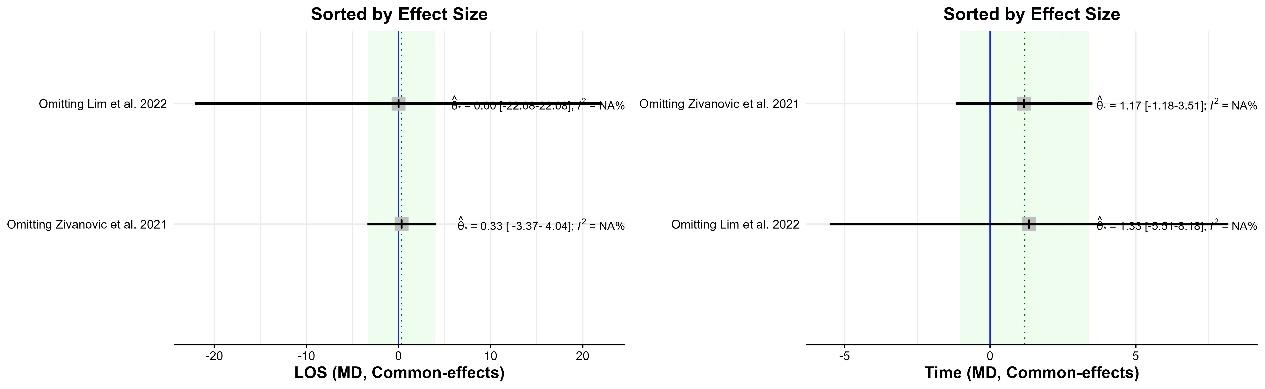


**Figure S3.** Forest plots showing the results of the leave-one-out sensitivity analysis for continuous outcomes (length of hospital stay and chemotherapy timing; number of studies: 2 (n=282)).
